# GEN-27, a Newly Synthetic Isoflavonoid, Inhibits the Proliferation of Colon Cancer Cells in Inflammation Microenvironment by Suppressing NF-*κ*B Pathway

**DOI:** 10.1155/2016/2853040

**Published:** 2016-02-23

**Authors:** Yajing Wang, Ping Lu, Weifeng Zhang, Qianming Du, Jingjing Tang, Hong Wang, Jinrong Lu, Rong Hu

**Affiliations:** ^1^State Key Laboratory of Natural Medicines, Department of Physiology, China Pharmaceutical University, Jiangsu, Nanjing 210009, China; ^2^Department of Organic Chemistry, China Pharmaceutical University, Jiangsu, Nanjing 210009, China

## Abstract

Nonresolving inflammation is one of the consistent features of the tumor microenvironment in the intestine and plays a critical role in the initiation and development of colon cancer. Here we reported the inhibitory effects of GEN-27, a new derivative of genistein, on the inflammation-related colon cancer cell proliferation and delineated the mechanism of its action. The results indicated that GEN-27 inhibited the proliferation of human colon tumor HCT116 cells stimulated by culture supernatants of LPS-induced human monocytes THP-1 cells and significantly decreased LPS-induced secretion of proinflammatory cytokines interleukin-6 and interleukin-1*β* in THP-1 cells. The HCT116 cell proliferation elicited by THP-1-conditioned medium could be blocked by the interleukin-1 receptor antagonist (IL-1RA). Further mechanistic study revealed that GEN-27 remarkably inhibited the nuclear translocation of NF-*κ*B and phosphorylation of I*κ*B and IKK*α*/*β* in both HCT116 and THP-1 cells. In addition, GEN-27 markedly suppressed the HCT116 cell proliferation stimulated by IL-1*β* treatment, which was dependent on the inhibition of NF-*κ*B/p65 nuclear localization, as verified by p65 overexpression and BAY 11-7082, an NF-*κ*B inhibitor. Taken together, our findings established that GEN-27 modulated NF-*κ*B signaling pathway involved in inflammation-induced cancer cells proliferation and therefore could be a potential chemopreventive agent against inflammation-associated colon cancer.

## 1. Introduction

Colorectal cancer (CRC) is one of the most common malignancies and the leading cause of cancer-related mortality among both men and women worldwide [1, 2]. Patients with inflammatory bowel disease (IBD), such as ulcerative colitis (UC) or Crohn's disease, have an increased risk of CRC [3]. The cumulative probability of CRC in UC patients ranges from 2% after 10 years of disease up to 18% after 30 years of disease [4].

It has been suggested that the leukocyte infiltrates exist in neoplastic tissue and there is a close association between chronic inflammation and cancer [5]. Chronic inflammation may be involved in all three stages of tumor development, which contributes to the tumor initiation by inducing DNA damage and chromosomal rearrangement or amplification. It also facilitates tumor promotion by inducing the formation of small clusters of malignant cells. Additionally, inflammation promotes tumor progression by inducing angiogenesis, invasion, and metastasis [6]. Overall, increasing evidence from experiments and epidemiological, preclinical, and clinical studies indicates that chronic inflammation is closely related to tumorigenesis, with CRC being one of the paradigms of the link between inflammation and cancer [7].

Inflammatory cytokines in tumor microenvironment regulate the communication between tumor and stromal cells, and tumor interactions with the extracellular matrix, thereby promoting tumor development [5]. Greten et al. found the evidence of cytokine-regulated tumor promotion in AOM/DSS mouse model of CAC [8]. Primary transcription factors such as nuclear factor *κ*B (NF-*κ*B) and signal transducer and activator of transcription 3 (STAT3), which are driven by inflammatory cytokines including tumor-necrosis factor *α* (TNF*α*), interleukin-6 (IL-6), and interleukin-1*β* (IL-1*β*), are key orchestrators controlling inflammation-related cancer [9].

NF-*κ*B plays a crucial role in the immediate-early pathogen responses and regulates many cellular processes including immune signaling, inflammation, cell proliferation, apoptosis, and cancer development. It is sequestered in the cytoplasm which forms an inactive complex with its inhibitor I*κ*B under basal conditions. Upon stimulation with corresponding ligands, such as LPS, IL-1*β*, or TNF*α*, the I*κ*B kinase (IKK) complex is activated, which leads to the phosphorylation and proteasomal degradation of I*κ*B, followed by translocation of NF-*κ*B into the nucleus to initiate specific target gene transcription [10]. Dysregulation of NF-*κ*B activation has been strongly related to several autoimmune diseases, such as rheumatoid arthritis, inflammatory bowel disease, multiple sclerosis, and type I diabetes. In addition, chronic exposure to inflammatory signals in the tumor microenvironment leads to NF-*κ*B activation in malignant cells, further driving tumor cells survival and proliferation. Thus NF-*κ*B pathway has attracted much attention due to its important role in inflammatory diseases and cancers.

Genistein (5,7-dihydroxy-3-(4-hydroxyphenyl)chromen-4-one), a isoflavonoid isolated from dietary soybean, has shown a wide variety of biological activities, such as antioxidant, anti-inflammatory, and anticancer properties, particularly in cancer prevention [11]. In CRC, previous studies have shown that genistein is capable of inducing G2/M phase cell cycle arrest and programmed cell death, inhibiting cell proliferation, and reducing metastasis [12, 13]. Its mechanisms include inhibition of topoisomerase I and topoisomerase II and DNA polymerase II and downregulation of genes encoding cyclins: B1 and D1 [14]. It also suppresses NF-*κ*B pathway, activates ATM/p53-p21 cross-regulatory network, and attenuates WNT signaling by upregulating sFRP2 protein [15, 16].

**Figure 1:**
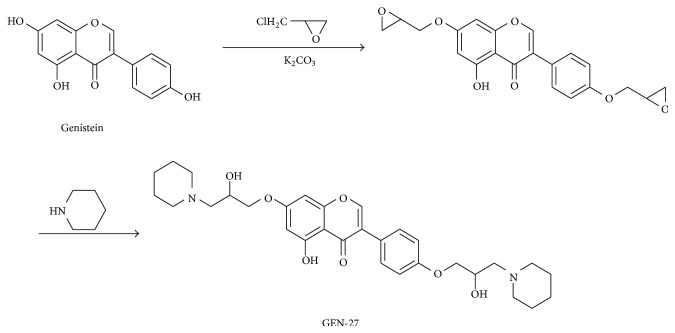
The synthetic route of GEN-27.

GEN-27 (5-hydroxy-7-[2-hydroxy-3-(piperidin-1-yl)propoxy]-3-{4-[2-hydroxy-3-(piperidin-1-yl)propoxy]phenyl}-4H-chromen-4-one), a newly synthesized derivative of genistein, was synthesized from genistein through two steps as indicated in Figure 1. Initially, the phenolic hydroxy groups at the C7 and C4′ of genistein were alkylated with (chloromethyl) ethylene oxide in dry ethanol in the presence of K_2_CO_3_. Then piperidines were coupled with the epoxy substrate to afford GEN-27. It was identified by IR, 1H-NMR, MS, and elemental analysis. The purity was 99.31% determined with HPLC (mp: 140–143°C).

Here in this study, we aimed to study the inhibitory effects of GEN-27 on the proliferation of human colorectal carcinoma HCT116 cells in the inflammatory microenvironment and the underlying mechanisms of the interaction between inflammatory cells and tumor cells.

## 2. Materials and Methods

### 2.1. Reagents and Antibodies

GEN-27 was obtained from College of Science, China Pharmaceutical University (Nanjing, China). GEN-27 (purity > 99.5%) was applied in DMSO to 0.1 M and stored at −20°C. The concentrations used here were 1, 5, 10, and 20 *μ*M in vitro and freshly diluted with DMEM to final concentration. LPS, [3-(4,5-dimethylthiazol-2-yl)-2,5-diphenyltetrazolium bromide] (MTT), and BAY 11-7082 were purchased from Sigma-Aldrich (St. Louis, Mo, USA). LPS was dissolved in distilled phosphate-buffered saline (PBS) at 10 mg/mL and stored in small aliquots at −20°C. Primary antibodies against NF-*κ*B p65 (C22B4), p-I*κ*B*α* (Ser32), I*κ*B*α*, p-IKK*α*/*β* (Ser176/180), and IKK*α* were obtained from Cell Signaling Technology (Danvers, MA); antibodies against cyclin D1 (L283), bcl-2 (P65), PCNA, *β*-actin, and goat anti-rabbit IgG (H&L) HRP were obtained from Bioworld Technology (St. Louis, MN). Recombinant human interleukin-1 receptor antagonist (IL-1RA) and recombinant human IL-1*β* were purchased from Genscript Corp. (Nanjing, China). Enzyme-linked immunosorbent assay (ELISA) kits for determining IL-6 and IL-1*β* were from Boster Biotech Co. Ltd. (Wuhan, China). Fetal bovine serum and RPMI-1640 were from Gibco (Grand Island, NY, USA).

### 2.2. Cell Lines and Culture Condition

Human colorectal cancer cell line HCT116 and human acute monocytic leukemia cell line THP-1 were purchased from the Cell Bank of Institute of Cell Biology (Shanghai, China). These two cells were cultured in RPMI-1640 medium supplemented with 10% heat-inactivated fetal bovine serum. Cells were maintained at 37°C in a humidified incubator containing 5% CO_2_.

### 2.3. Colorimetric MTT Assay

The cytotoxicity was measured by the modified MTT assay. Briefly, the logarithmic cells were plated into 96-well plates at a density of 4000~5000 cells/well in a final volume of 100 *μ*L medium for 12 h at 37°C and then treated with various concentrations of GEN-27 at indicated durations. After 24 h or 48 h incubation, the absorbance (A) was measured at 570 nm by the Universal Microplate Reader (ELx800, BioTek Instruments Inc., Winooski, VT). Percentage of cytotoxicity was determined as follows: percentage of cytotoxicity = [1 − (A_570_ of test sample)/(A_570_ of control sample)] × 100%. The IC_50_ was taken as the concentration that caused 50% inhibition of cell proliferation and was calculated by SAS statistical software. All assays were performed in triplicate.

### 2.4. Cell Cycle and Apoptosis Assay

HCT116 cells were plated into 6-well tissue culture plates at approximately 2 × 10^5^/well and treated with various concentrations of GEN-27. After incubation, they were harvested and resuspended with PBS. Apoptosis-mediated cell death of tumor cells was examined using double staining with recombinant FITC-conjugated Annexin-V and propidium iodide (PI), according to the manufacturer's protocol of the Annexin-V-FITC Apoptosis Detection Kit (KeyGen, Nanjing, China). For cell cycle assay, cells were trypsinized, washed with PBS, and fixed in 1.5 mL 95% ethanol at 4°C overnight followed by incubation with RNase and staining by PI. Data acquisition was performed with FACSCalibur flow cytometry (Becton Dickinson, San Jose, CA, USA).

### 2.5. Total RNA Isolation and Real-Time PCR

Human monocyte THP-1 cells were incubated with different concentrations of GEN-27 in the presence or absence of LPS (10 *μ*g/mL). After incubation for 24 h, total RNA was isolated using Trizol Reagent (Invitrogen, Carlsbad, CA, USA) according to the manufacturer's protocol. Then the concentration and purity of total RNA were measured by the ratio of A_260_/A_280_ using an Epoch Microplate Spectrophotometer (BioTek, USA). Real-time PCR was performed as follows: RNA samples were reverse transcribed to cDNA and subjected to quantitative PCR, which was performed with the LightCycler^®^ 96 Real-Time PCR System (Roche, Basel, Swiss) using AceQ qPCR SYBR Green Master Mix (Vazyme, Nanjing, China). The program for amplification was 1 cycle of 95°C for 2 min followed by 40 cycles of 95°C for 10 s, 60°C for 30 s, and 95°C for 10 s. Glyceraldehyde-3-phosphate dehydrogenase (GAPDH) was used as a loading control in the analytical gels. Primer sequences used in this study were listed as follows:

 IL-6: 5′-TGTAGTGAGGAACAAGCCAGAG-3′ (forward), 5′-TACATTTGCCGAAGAGCC-3′ (reverse);
 IL-1*β*: 5′-AGGCTGCTCTGGGATTC-3′ (forward), 5′-GCCACAACAACTGACGC-3′ (reverse);
 GAPDH: 5′-AAGGTCGGAGTCAACGGATTT-3′ (forward), 5′-AGATGATGACCCTTTTGGCTC-3′ (reverse).

### 2.6. Enzyme-Linked Immunosorbent Assay (ELISA)

For analysis of cytokine production, THP-1 cells were cultured at 1 × 10^5^ cells/mL for 24 h. Cells were centrifuged at 2,000 rpm at 4°C for 10 min and the supernatants were carefully collected and applied onto the precoated human IL-6 or IL-1*β* microplate. ELISAs were conducted according to the manufacturer's recommendations. All reactions were performed in triplicates and the experiments were repeated three times for statistical analysis. Levels of cytokines were expressed in ng/mL.

### 2.7. Preparation of Cytosolic and Nuclear Extracts and Whole Cell Lysates

HCT116 or THP-1 cells were cultured to 70% confluence and then treated with LPS (10 *μ*g/mL) alone or in combination with GEN-27 for indicated times. Following treatments, cells were harvested by centrifugation and then washed with ice-cold PBS three times. Whole cell lysates were obtained according to the method as described in the following: prepared cells were lysed on ice for 1 h in lysis buffer (100 mM of Tris-Cl; pH 6.8, 4% (m/v) SDS; 20% (v/v) glycerol; 200 mM of *β*-mercaptoethanol; 1 mM of PMSF; 0.1 mM of NaF and 1 *μ*M DTT). The lysates were clarified by centrifugation at 12,000 rpm for 20 min at 4°C, and the supernatant was collected. The isolation of cytosolic and nuclear extracts was performed according to the method of Nuclear-Cytosol Extraction Kit (KeyGEN Biotech, China) with more modification. Specific steps are as follows: after washing, prepared cells were lysed with membrane lysis buffer (10 mM Hepes-PH 8.0, 10 mM KCl, 1.5 mM MgCl_2_, and 1 *μ*M DTT), incubated for 15 min on ice, and then added to 1% NonidetP-40 (NP-40) for 10 sec; the supernatant was collected as cytoplasmic fractions after centrifugation at 13,000 rpm for 20 min at 4°C. The precipitate was added with nuclear lysis buffer (2 mM Hepes-PH 8.0, 1.5 mM MgCl_2_, 42 *μ*M NaCl, 1 *μ*M DTT, 25 *μ*L glycerol, and 0.2 *μ*M EDTA) for 1 h on ice and vortexed every 10 min. The concentration of protein was detected using BCA assay with a Varioskan multimode microplates spectrophotometer (Thermo, Waltham, MA).

### 2.8. Western Blot Analysis

For immunoblot analysis, equal amounts of protein samples (40~60 *μ*g) were separated electrophoretically using 10% sodium dodecyl sulfate-polyacrylamide gel electrophoresis (SDS-PAGE) under reducing conditions. The gels were then transferred to 0.45 *μ*m polyvinylidene difluoride (PVDF) membranes (Millipore, Bedford, MA) using a semidry transfer system (Bio-Rad, Hercules, CA). The membranes were blocked for nonspecific binding with 3% bovine serum albumin (BSA) in PBS for 90 min at 37°C. The blots were incubated with specific primary antibodies overnight at 4°C. After being washed with PBST three times, the blots were incubated with horse radish peroxidase- (HRP-) conjugated immunoglobulin G (IgG) for 1 h at 37°C, and chemiluminescence was detected with Pierce ECL Western blotting substrate (Thermo Scientific) and visualized by ChemiDoc MP Imaging system (Bio-Rad, Hercules, CA, USA).

### 2.9. Immunofluorescence

For detection of NF-*κ*B p65 translocation to the nuclear, HCT116 cells were planted at 1~2 × 10^5^ cells/mL on glass cover slips in 6-well plates and treated with 10 *μ*M GEN-27 for the indicated time period with or without LPS (10 *μ*g/mL). After treatments, cells were washed twice with PBS and fixed in 4% paraformaldehyde (PFA) at room temperature for 20 min. After washing with PBS, cells were permeabilized with 0.2% Triton X-100 at 4°C for 10 min, blocking (PBS containing 3% BSA for 1 h) was followed by washing thrice with PBS for 5 min, and then the cells were incubated with the primary antibody NF-*κ*B p65 at a dilution of 1 : 200 in PBS containing 0.5% BSA at 37°C overnight. The next day, cells were washed three times with PBS followed by incubating with Green-Fluorescence Alexa Fluor 488 dye labeled donkey anti-rabbit IgG antibody for 1 h at 37°C. After the immunoreactions, the cover slips were mounted onto microscope slides using Ultra Cruz*™* Mounting Medium (Santa Cruz Biotechnology Inc., CA). Immunofluorescence photomicrographs were captured using fluorescent microscope (Olympus IX51, Olympus Corporation, Tokyo, Japan).

### 2.10. Culture of Human Colon Cancer HCT116 Cells with Conditioned Media from LPS-Treated Human Monocytes THP-1 Cells

HCT116 cells were seeded into 96-well plates at a density of 4000~5000 cells/well in 100 *μ*L medium, grown to 60~70% confluence one day before treatment. THP-1 cells were cultured in 6-well plates and then stimulated with LPS (10 *μ*g/mL) combination of various concentrations of GEN-27 (1, 5, and 10 *μ*M). After the cells were collected by centrifugation, the cultured supernatant was aseptically stored at 4°C for use. Prepared HCT116 cells were (1) left untreated or treated with (2) 10 *μ*g/mL of LPS only, (3) various concentrations of GEN-27 (1, 5, and 10 *μ*M), and (4) cultured supernatant from THP-1 cells which were processed as described above. After HCT116 cells were cultured for 24 h, the cell culture supernatant was removed and the proliferation was determined by MTT assay as described above.

### 2.11. Plasmids Transfection

NF-*κ*B/p65 plasmid and control plasmid (Addgene, Cambridge, MA, USA) transfections were performed according to the manufacturer's instructions of ExFectTM Transfection Reagent (Vazyme Biotech). The extent of gene overexpression was determined by Western blot.

### 2.12. Statistical Analyses

Data were expressed as means ± SDs from triplicate experiments performed in a parallel manner unless otherwise indicated. Statistical significance was done using an analysis of variance that was followed by Student's *t*-test and Newman-Keuls test. ^*∗*^ *P* < 0.05 and ^*∗∗*^ *P* < 0.01 were considered to be statistically significant.

## 3. Results

### 3.1. GEN-27 Inhibits the Cell Viability of THP-1 Cells

**Figure 2:**
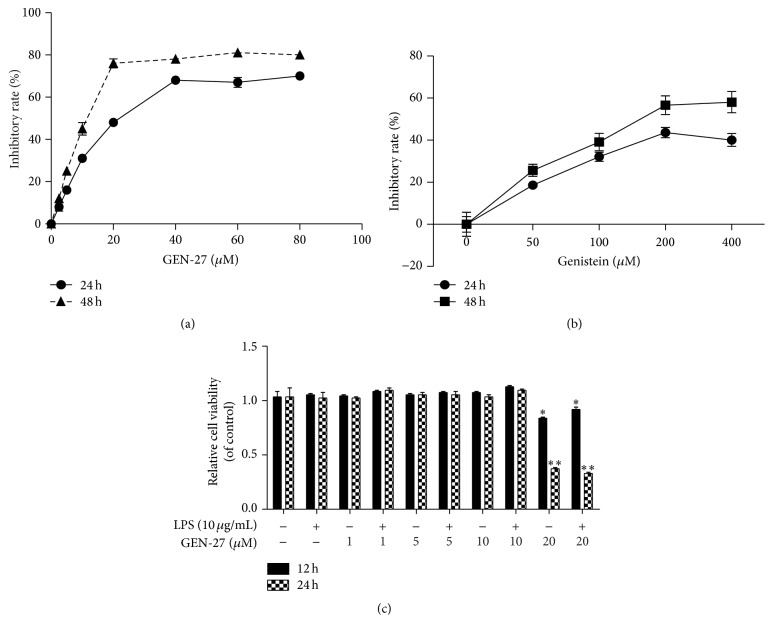
Effects of GEN-27 and LPS on the cell viability of THP-1 cells. (a) THP-1 cells were treated with the different concentrations of GEN-27 alone or (b) the different concentrations of genistein. (c) THP-1 cells were exposed to GEN-27 at different concentrations (1, 5, 10, and 20 *μ*M) in the presence or absence of LPS (10 *μ*g/mL). After treatments, cell viability was determined by MTT assay. Each value indicates the means ± SDs and is representative of the results obtained from three independent experiments. Asterisks (^*∗*^ *P* < 0.05 versus control group; ^*∗∗*^ *P* < 0.01 versus control group, and ^#^ *P* < 0.05 versus LPS group) indicate significant difference compared with the appropriate control cells.

Initially, we determined the cytotoxicity of GEN-27 in THP-1 cells using MTT assay. As shown in Figure 2(a), GEN-27 inhibited the growth of THP-1 cells with IC_50_ values of 24.49 ± 0.21 *μ*M (24 h) and 11.28 ± 0.26 *μ*M (48 h), compared with its parent compound genistein with an IC_50_ of 192.4 ± 2.28 *μ*M at 48 h (Figure 2(b)). Combined with LPS treatment, 1, 5, and 10 *μ*M of GEN-27 exerted no effect on the survival and proliferation of THP-1 cells. However, GEN-27 at 20 *μ*M obviously reduced the cell viability of THP-1 cells (Figure 2(c)). Therefore, 1, 5, and 10 *μ*M of GEN-27 were used for all subsequent experiments.

### 3.2. GEN-27 Inhibits Proliferation of Human Colorectal Carcinoma HCT116 Cells

**Figure 3:**
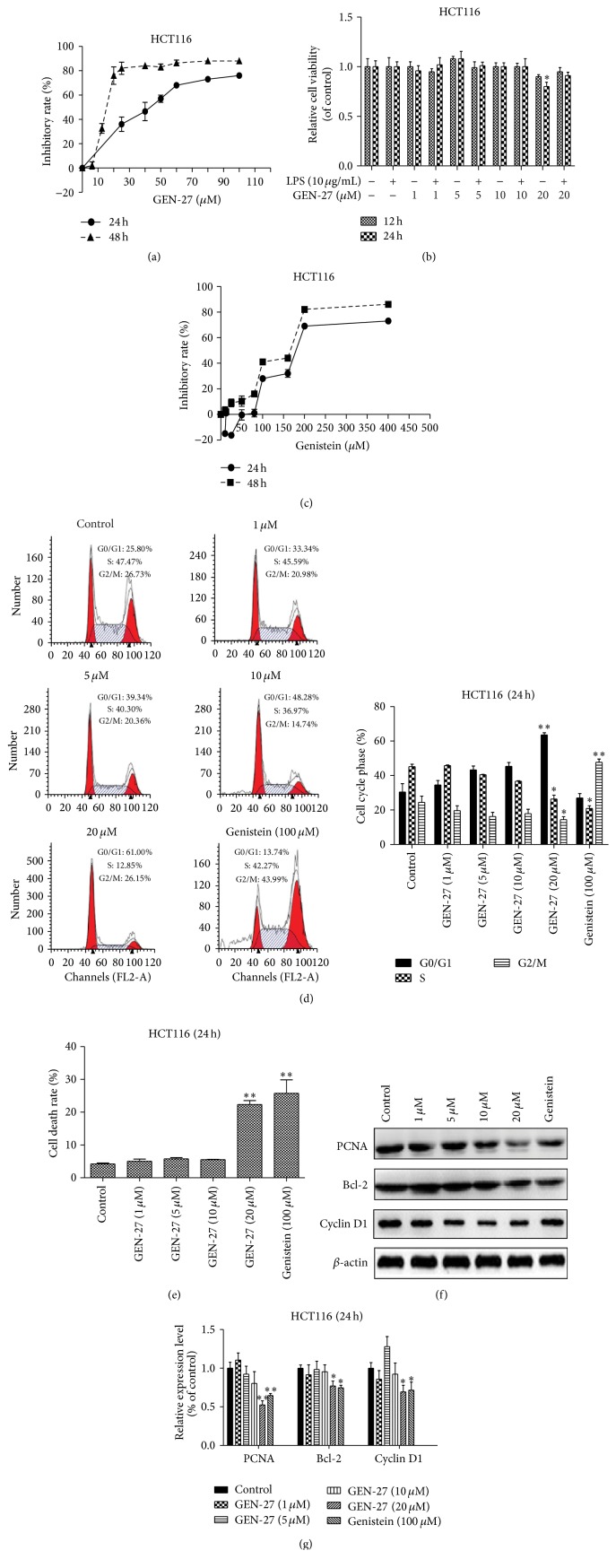
Effects of GEN-27 and LPS on the cell viability of HCT116 cells. (a) Cells were treated with GEN-27 for 24 h and 48 h, (b) exposed to GEN-27 in the presence or absence of LPS (10 *μ*g/mL), or (c) exposed to different concentrations of genistein; then cell viability was determined by the MTT assay. (d) Cell cycle was detected by flow cytometry following PI staining. Cells were treated with different concentrations of GEN-27 and 100 *μ*M genistein for 24 h. Different percentages of three cell phases (G0/G1, S, and G2/M) were shown. (e) Annexin-V/PI double-staining assay of HCT116 cells. Cells were treated with the indicated doses of GEN-27 for 24 h; histograms of death rates were quantitated, containing the early and late apoptosis. (f) The expressions of total proteins PCNA, bcl-2, and cyclin D1 were assessed by Western blot. (g) The relative expressions of total proteins PCNA, bcl-2, and cyclin D1 were normalized to *β*-actin. Each value indicates the means ± SDs and is representative of the results obtained from three independent experiments. ^*∗*^ *P* < 0.05 and ^*∗∗*^ *P* < 0.01 compared with control.

As shown in Figure 3(a), GEN-27 dramatically reduced the cell viability in HCT116 cells with IC_50_ values of 37.98 ± 0.13 *μ*M (24 h) and 15.11 ± 0.80 *μ*M (48 h), respectively. However, genistein exhibited relatively weak inhibitory effect on the proliferation of HCT116 cells, with IC_50_ of 189.3 ± 2.27 *μ*M (24 h) and 151 ± 2.13 *μ*M (48 h) (Figure 3(c)). Consistent with what we found in THP-1 cells, 1, 5, and 10 *μ*M GEN-27 plus LPS did not induce evident cell death in HCT116 cells (Figure 3(b)). Moreover, different from genistein (100 *μ*M), which showed a dramatic G2/M phase arrest, GEN-27 dose-dependently increased the G0/G1 population in HCT116 cells (Figure 3(d)). The apoptosis-induced cell death rate was significantly elevated by GEN-27 treatment, as determined by Annexin-V/PI assay. The data from Western blot demonstrated that GEN-27 dose-dependently reduced the expression levels of proliferating cell nuclear antigen (PCNA), apoptosis-associated protein bcl-2, and cell cycle regulation protein cyclin D1 (Figures 3(f) and 3(g)). Taken together, GEN-27 inhibits HCT116 cell proliferation through inducing G0/G1 cell cycle arrest and cell apoptosis.

### 3.3. GEN-27 Suppresses the Proliferation of HCT116 Cells in Response to THP-1-Conditioned Medium Induced by LPS

**Figure 4:**
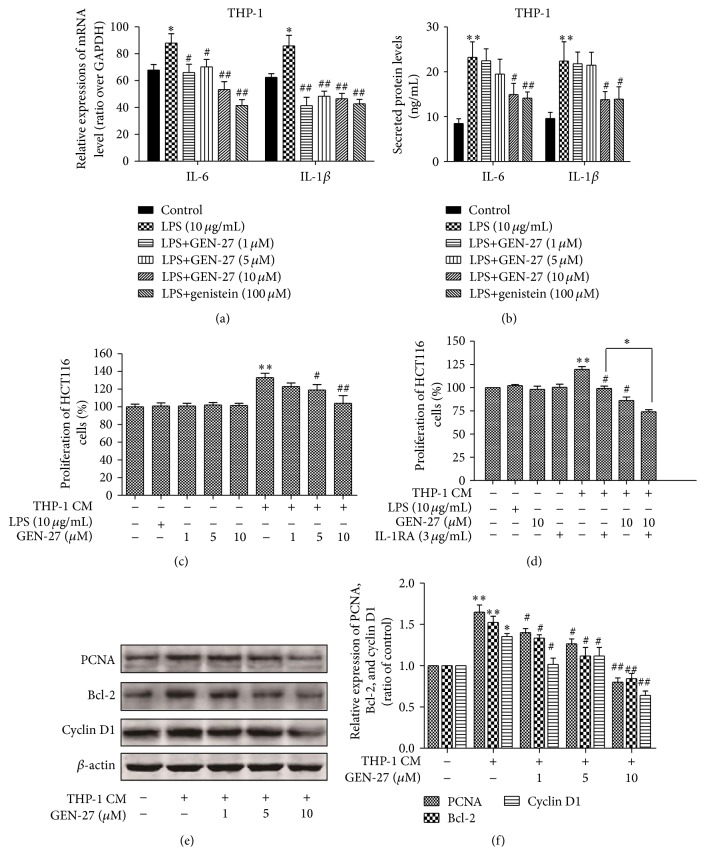
Effects of GEN-27 on LPS-induced production of proinflammatory cytokines in THP-1 cells and the proliferation of HCT116 cells in response to the stimulation by THP-1-conditioned medium. (a) Human THP-1 cells were incubated with 10 *μ*g/mL LPS for 24 h in the absence or presence of 1, 5, and 10 *μ*M GEN-27 and 100 *μ*M genistein. The expression of IL-6 and IL-1*β* mRNA in THP-1 cells was assessed by real-time PCR. GAPDH was used as an endogenous housekeeping gene. (b) The secretion levels of IL-6 and IL-1*β* in THP-1 cells after treatments of indicated doses of GEN-27 and genistein were assessed by ELISA. (c and d) HCT116 cells were either left untreated or treated with 10 *μ*g/mL LPS, or THP-1-conditioned medium and indicated dose of GEN-27 together, or combination of 3 *μ*g/mL IL-1RA, THP-1-conditioned medium, and indicated dose of GEN-27 for 24 h. Cell viability was assessed using an MTT assay and the results are expressed as the percentage of surviving cells over control cells. (e and f) The expressions of total proteins PCNA, bcl-2, and cyclin D1 were assessed by Western blot. The relative expressions of total proteins PCNA, bcl-2, and cyclin D1 were normalized to *β*-actin. Each value indicates the means ± SDs and is representative of the results obtained from three independent experiments. ^*∗*^ *P* < 0.05 and ^*∗∗*^ *P* < 0.01 compared with control; ^#^ *P* < 0.05 and ^##^ *P* < 0.01 versus LPS alone.

The interaction between tumor cells and multiple components of the tumor microenvironment, including B and T cells, macrophages, mast cells, fibroblasts, and extracellular matrix, could promote tumor progression [9]. These components can regulate cell growth, differentiation, and survival of tumor cells and thus contribute to tumor promotion and progression via producing soluble factors such as chemokines, cytokines, and growth factors. THP-1 cells have a uniform genetic background with peripheral blood mononuclear cells (PBMC). In response to stimulation with LPS, THP-1 cells exhibit a similar transcriptional pattern with PBMC-derived macrophages [17]. Thus THP-1 cells are widely used to mimic monocytes in cell culture models. Proinflammatory factors IL-6 and IL-1*β* are secreted by many cell types, such as immune cells and tumor, stromal, and endothelial cells, which play an important role in inflammation-associated carcinogenesis [3].  Figure 4(b) showed that LPS (10 *μ*g/mL) treatment stimulated the secretion of the IL-6 and IL-1*β* from THP-1 cells, and GEN-27 dramatically reduced this increase stimulated by LPS in a dose-dependent manner. Consistently, real-time PCR data revealed that the mRNA levels of IL-6 and IL-1*β* increased by LPS were significantly downregulated by the treatment of GEN-27 in a dose-dependent manner (Figure 4(a)).

To determine the effects of inflammatory cells on tumor cells, HCT116 cells were cultured with the culture supernatant of THP-1 cells stimulated by LPS for 24 h. As shown in Figure 4(c), THP-1 cell-derived factors enhanced the proliferation of HCT116 cells and this effect was suppressed by GEN-27 treatment in a dose-dependent manner, as verified by the reduction of the expressions of PCNA, cyclin D1, and bcl-2 proteins using Western blot (Figures 4(e) and 4(f)). As shown in Figure 4(d), 3 *μ*g/mL IL-1 receptor antagonists (IL-1RA) or 10 *μ*M GEN-27 treatment alone significantly suppressed the proliferation of HCT116 cells induced by conditioned medium, while the suppression induced by GEN-27 was not affected by IL-1RA. Compared with the IL-1RA treatment alone, cotreatment of GEN-27 plus IL-1RA exhibited a significant additive effect on the reduction of HCT116 cell proliferation, which confirmed the vital role of IL-1*β* in the anticancer effect of GEN-27. Taken together, GEN-27 significantly inhibited HCT116 cells proliferation stimulated by THP-1-derived conditioned medium via reducing the secretion of IL-6 and IL-1*β* from THP-1 cells.

### 3.4. GEN-27 Inhibits LPS-Induced NF-*κ*B Pathway in THP-1 Cells

**Figure 5:**
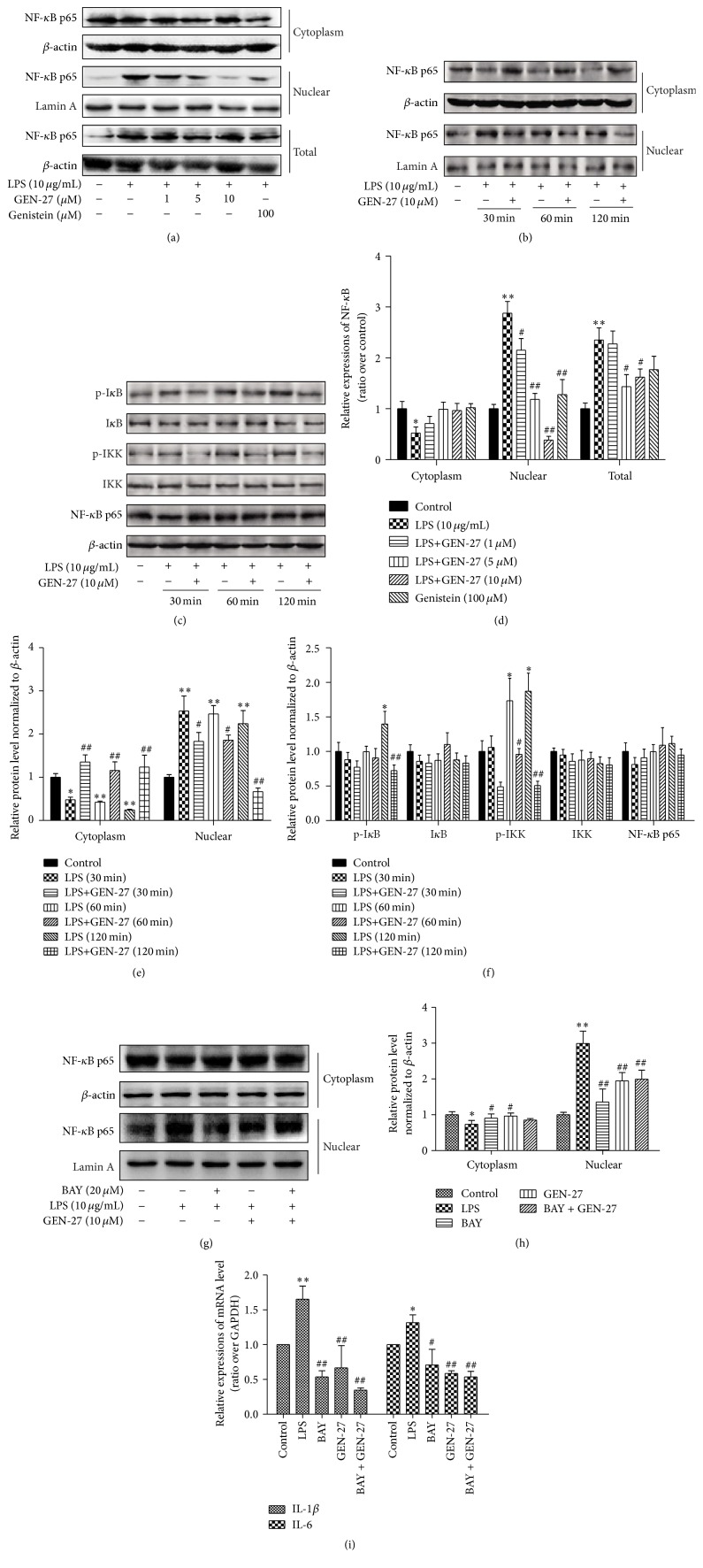
GEN-27 inhibited LPS-induced NF-*κ*B p65 activation in THP-1 cells. THP-1 cells were either left untreated or treated with 10 *μ*g/mL LPS, or 10 *μ*g/mL LPS, and indicated concentrations of GEN-27 together, or combination of 10 *μ*g/mL LPS, 10 *μ*M GEN-27, and 20 *μ*M Bay 11-7082. (a, b, and g) The expressions of NF-*κ*B p65 in the cytoplasm and nucleus were determined by Western blot analysis. (c) The expressions of total NF-*κ*B p65, p-I*κ*B*α*, I*κ*B*α*, p-IKK*α*/*β*, and IKK*α*/*β* were determined by Western blot analysis, respectively. (d, e, f, and h) The relative expressions of NF-*κ*B p65, p-I*κ*B*α*, I*κ*B*α*, p-IKK*α*/*β*, and IKK*α*/*β* were normalized to *β*-actin. (i) The mRNA expressions of IL-6 and IL-1*β* in THP-1 cells from each group were determined by real-time PCR. Data (means ± SDs) were representative of at least three independent experiments. ^*∗*^ *P* < 0.05 and ^*∗∗*^ *P* < 0.01 compared with control; ^#^ *P* < 0.05 and ^##^ *P* < 0.01 versus LPS alone or corresponding LPS group at indicated time.

Previous studies have shown that NF-*κ*B is a crucial transcription factor that regulates the production of proinflammatory cytokines IL-6 and IL-1*β* [18, 19]. Translocation of p65, the functional active subunit of NF-*κ*B, is a hallmark of molecular inflammatory phenomenon. The results in Figures 5(a)–5(f) showed that LPS treatment caused a rapid translocation of NF-*κ*B p65 into nuclear fraction, which was markedly inhibited by GEN-27 in dose- and time-dependent manner. Meanwhile, the increase in total NF-*κ*B p65 expression induced by LPS was time- and dose-dependently decreased by GEN-27 in THP-1 cells (Figures 5(a) and 5(c)). One of the main mechanisms involved in the activation of NF-*κ*B is the phosphorylation of I*κ*B*α* and IKK*α*/*β*, which causes the accumulation of NF-*κ*B p65 and its translocation into the nucleus. As shown in Figures 5(c) and 5(f), LPS-treated THP-1 cells exhibited increased phosphorylation of I*κ*B*α* and IKK*α*/*β* and this induction was inhibited by GEN-27. To further identify the inhibitory effect of GEN-27 on NF-*κ*B signaling, BAY 11-7082, an NF-*κ*B inhibitor, was used to inhibit IKK*α*/*β* activation, which leads to the translocation of p65 into nucleus. LPS increased p65 level in nuclear fraction, which was repressed by GEN-27 or BAY 11-7082. The reduction induced by GEN-27 was not effected by BAY 11-7082, as verified by the expression of downstream target genes IL-6 and IL-1*β* at mRNA level (Figures 5(g)–5(i)). These findings suggested that GEN-27 suppressed NF-*κ*B activation by inhibiting nuclear translocation of NF-*κ*B p65 and the phosphorylation of IKK*α*/*β* and I*κ*B*α*.

### 3.5. GEN-27 Blocks LPS-Induced NF-*κ*B Pathway in HCT116 Cells

**Figure 6:**
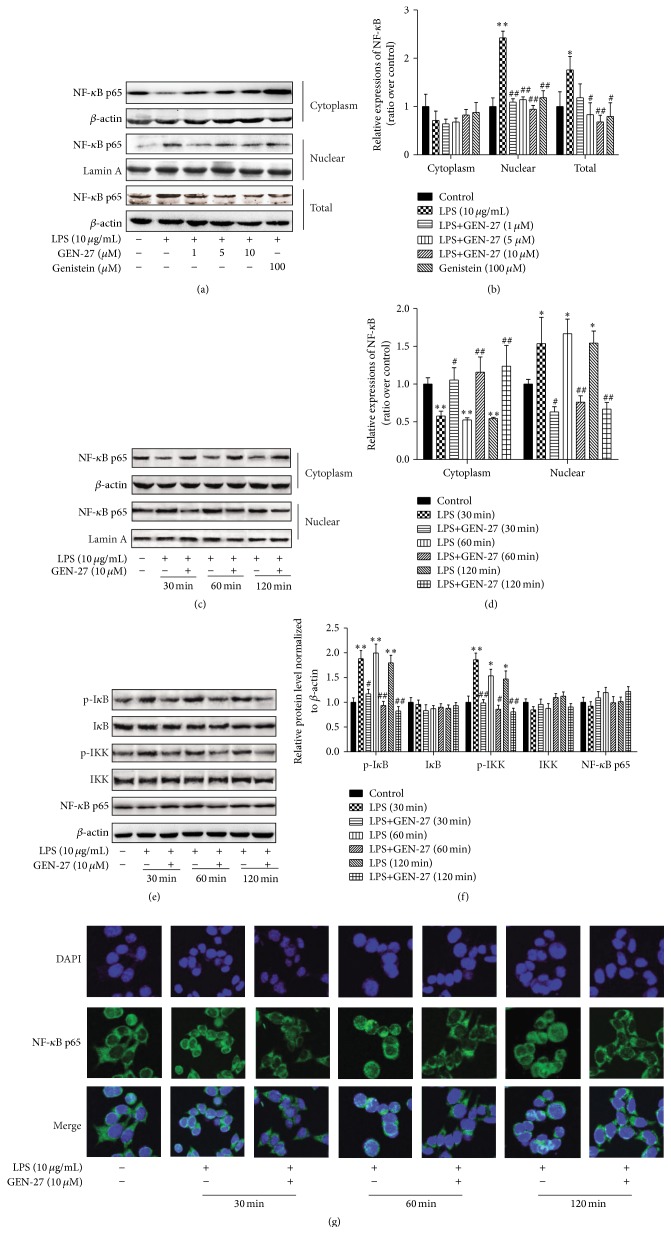
GEN-27 suppressed LPS-induced NF-*κ*B p65 activation in HCT116 cells. HCT116 cells were treated with LPS (10 *μ*g/mL) together with indicated concentrations of GEN-27 and genistein (100 *μ*M) for the indicated times. (a–f) The nuclear translocation and protein levels of NF-*κ*B/p65, p-I*κ*B*α*, I*κ*B*α*, p-IKK*α*/*β*, and IKK*α*/*β* were determined by Western blot. Data shown are representative of three experiments. The relative expressions of total proteins p-I*κ*B*α*, I*κ*B*α*, p-IKK*α*/*β*, and IKK*α*/*β* were normalized to *β*-actin. (i) The localization of NF-*κ*B p65 was visualized using fluorescence microscopy after immunofluorescence staining with NF-*κ*B p65 antibody (green). Cells were also stained with DAPI for visualization of the nuclei (blue). Data (means ± SDs) were representative of at least three independent experiments. ^*∗*^ *P* < 0.05 and ^*∗∗*^ *P* < 0.01 compared with control; ^#^ *P* < 0.05 and ^##^ *P* < 0.01 versus LPS alone or corresponding LPS group at indicated time.

To further corroborate the inhibitory effect of GEN-27 on NF-*κ*B pathway in HCT116 cells, we evaluated GEN-27's effects on NF-*κ*B activation induced by LPS. As shown in Figures 6(a)–6(d), the amount of NF-*κ*B p65 in the nucleus was markedly increased after exposure to LPS, and this response was significantly inhibited by GEN-27, which was validated by the reduction of the phosphorylation of I*κ*B*α* and IKK*α*/*β* (Figures 6(e) and 6(f)). The inhibition on the nuclear translocation of p65 by GEN-27 was further verified by immunofluorescence confocal microscopy (Figure 6(g)).

### 3.6. GEN-27 Inhibits IL-1*β*-Induced Cell Proliferation in HCT116 Cells

**Figure 7:**
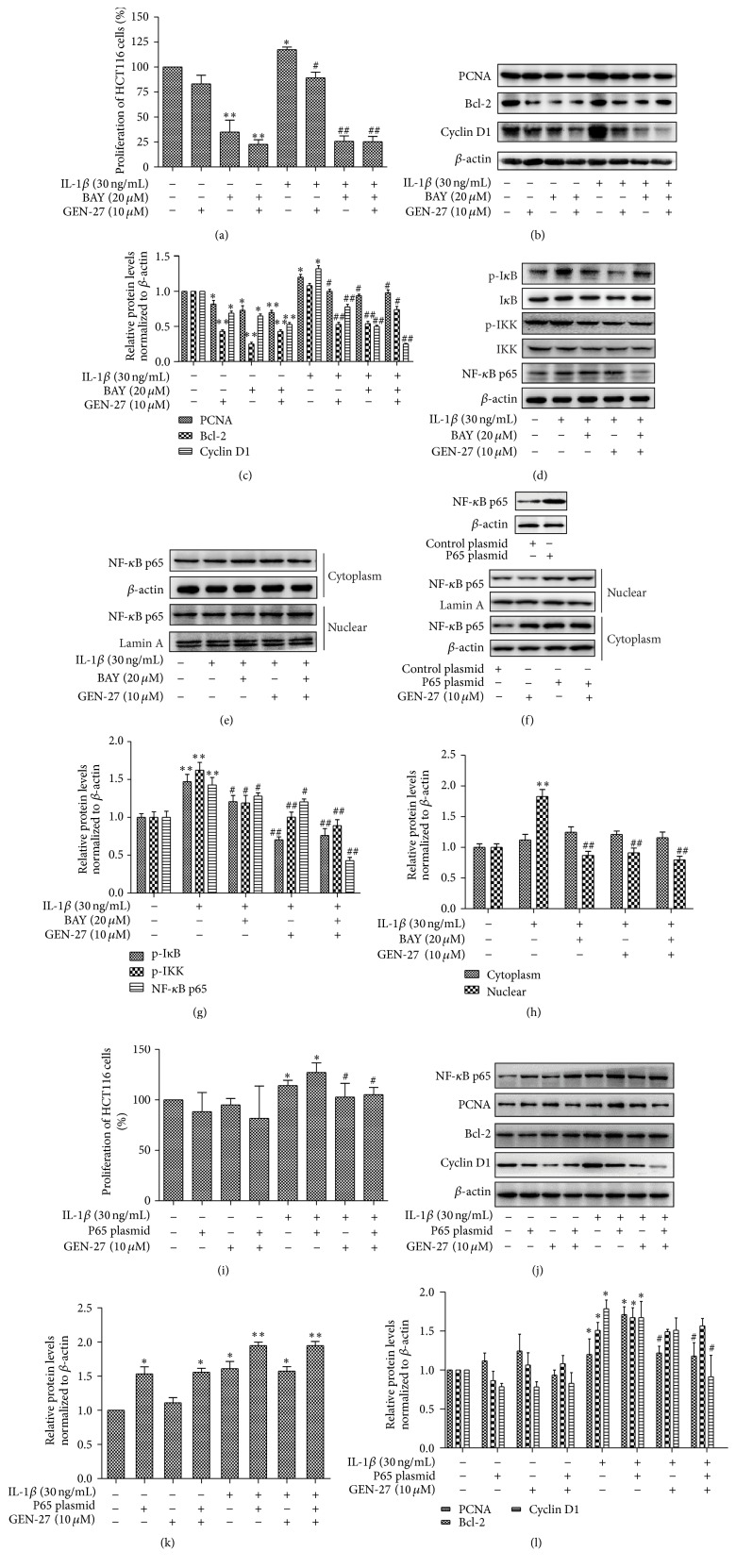
GEN-27 inhibited IL-1*β*-induced proliferation of human colon cancer cells. HCT116 cells were either left untreated or treated with 30 ng/mL IL-1*β*, or 30 ng/mL IL-1*β* and 10 *μ*M GEN-27 together, or combination of 30 ng/mL IL-1*β*, 10 *μ*M GEN-27, and 20 *μ*M Bay 11-7082 for 24 h. (a and i) Cell viability was assessed using an MTT assay and the results are expressed as the percentage of surviving cells over control cells. (b, d, and e) NF-*κ*B/p65 nuclear translocation and protein levels of total NF-*κ*B/p65, p-I*κ*B*α*, I*κ*B*α*, p-IKK*α*/*β*, IKK*α*/*β*, PCNA, bcl-2, and cyclin D1 were determined by Western blot. (c, g, and h) The quantitation of those proteins expression levels relative to *β*-actin expression according to (b, d, and e). (f) NF-*κ*B/p65 nuclear translocation and total protein levels of p65 with or without p65 overexpression. (j, k, and l) Protein levels of total NF-*κ*B/p65, PCNA, bcl-2, and cyclin D1 were determined by Western blot. The relative expressions of those proteins were normalized to *β*-actin. All graphic data shown are the means ± SDs. Results are representative of those obtained from three independent experiments. ^*∗*^ *P* < 0.05 and ^*∗∗*^ *P* < 0.01 compared with control; ^#^ *P* < 0.05 and ^##^ *P* < 0.01 versus IL-1*β* alone.

IL-1*β* is a pleiotropic proinflammatory cytokine and can be secreted by immune, stromal, and tumor cells. The interaction between colon cancer cells and inflammatory cells promotes secretion of the release of IL-1*β* from immune cells [20]. Elevated IL-1*β* levels have been shown to be associated with increased colon tumor growth and invasion [21]. As shown in Figure 7(a), IL-1*β* treatment caused the proliferation of HCT116 cells, which was blocked by GEN-27 or BAY 11-7082. GEN-27-mediated attenuation of cell proliferation was not changed by the cotreatment of BAY 11-7082, which was verified by the reduction in the expression levels of PCNA, bcl-2, and cyclin D1 (Figures 7(b) and 7(c)). Moreover, GEN-27 significantly repressed LPS-induced p65 nuclear localization and phosphorylation levels of I*κ*B*α* and IKK*α*/*β* (Figures 7(d) and 7(e)), which demonstrated that the antiproliferation effect of GEN-27 is dependent on the downregulation of NF-*κ*B pathway. To further corroborate this effect, HCT116 cells were transfected with p65 overexpression plasmid. The reduction of p65 nuclear localization induced by GEN-27 was remarkably reversed by p65 overexpression (Figure 7(f)). Moreover, overexpressed p65 did not influence the proliferation of HCT116 cells. Compared with p65 overexpression cells, the combined treatment with p65 plasmid and GEN-27 showed no effect on HCT116 cell proliferation (Figure 7(i)). However, when HCT116 were simultaneously treated by IL-1*β*, overexpressed p65 increased the cells proliferation and this effect was partly reversed by GEN-27 (Figure 7(i)), which were verified by the expression levels of PCNA, bcl-2, and cyclin D1 (Figures 7(j) and 7(l)). Taken together, these results indicated that GEN-27 inhibited IL-1*β*-induced proliferation of human colon cancer cells through blocking NF-*κ*B pathway.

## 4. Discussion

It is generally accepted that at least 15% of cancer is caused by chronic inflammation [22]. Chronic inflammation has been proposed to be a major contributor to CRC, which is the third leading cause of cancer-related death in developed countries. In fact, the CRC incidence is relatively low in Asian countries compared with Western countries. Lower incidence and mortality rates of CRC have been thought to be due to high consumption of soybeans and their products in Asian countries [23–25]. Genistein is one of the major bioactive constituents of soybeans and exerts antioxidant, anti-inflammatory, anticancer, antiviral, and neuroprotective activities. In this study, we initially investigated the inhibitory effect of GEN-27, a genistein derivative, on human monocyte THP-1 cells and colon cancer HCT116 cells and found that GEN-27 inhibited cell proliferation and induced G0/G1 cell cycle arrest and cell apoptosis with higher potency than its parent compound genistein. The main aim of this study was to determine the effects of GEN-27 on the proliferation of colon cancer cells in inflammatory microenvironment. We utilized LPS-stimulated THP-1 cells to mimic the inflammatory cells in microenvironment of solid tumors. Since GEN-27-mediated reduction of cell growth would influence the observation of its anti-inflammatory effect, we chose relatively low concentrations that were nontoxic to cells in subsequent experiments.

Previous studies have reported that genistein could suppress cell growth and proliferation in multiple cancer cell lines by an accumulation of cells at the G2/M phase. This effect was related to the inhibition of insulin-like growth factor-1 (IGF-1) receptor signaling and the PI3k/AKT pathway, also including the upregulation of p53 and CDK inhibitor p21 waf1/cip1 [12, 26–30]. However, several reports found that genistein induces G0/G1 arrest in MCF-7 cells, HB4a cells, BALB/c 3T3 cells, and B16-F1 cells mediated through induction of p21 and suppression of cyclin D1 and cyclin E, key protein regulators of G1/S transition of cell cycle [31–33]. In our study, 100 *μ*M genistein delayed HCT116 cells at G2/M phase, but its derivative GEN-27 (10 *μ*M) induced G0/G1 arrest through inhibition of cyclin D1 expression and NF-*κ*B nuclear translation. Activated NF-*κ*B can upregulate the transcription of cell cycle regulator cyclin D1 via binding to multiple sites within the promoter region, which promotes the G1/S-phase transition. During cell cycle progression, cyclin D1 activates cyclin-dependent kinases CDK4 and CDK6 and then forms cyclin D1-CDK4 and D1-CDK6 complexes, which can phosphorylate the retinoblastoma protein, such as pRB and pRB-related p107 and p130 proteins. Phosphorylation of pRB, p107, and p130 derepresses the transcriptional activity of E2F transcription factors, thereby allowing the G1 to S-phase transition [34]. In addition, cyclin D1-CDK4/6 complexes sequester the cell cycle inhibitors p27Kip1 and p21Cip1 away from cyclin E-CDK2, thereby contributing to activation of cyclin E-CDK2 kinase. Therefore, the reduction of cyclin D1 could explain, by inhibiting NF-*κ*B nuclear translocation and I*κ*B phosphorylation, at least in part, the increase of cells in the G1/G0 phase by GEN-27 treatment in our experiments.

Many clinical studies depicted that most solid tumors infiltrated with immune cells, which promoted tumor progression. It had been shown that coculture of cancer cells with fibroblasts could generate an activated microenvironment, rich in inflammatory mediators and growth factors [35], or with macrophages could promote the release of IL-1*β*, which induced the activation of WNT signaling and supported the growth of tumor cells [20, 36]. In present study, similar results were observed where THP-1-derived conditional medium stimulated by LPS could promote the growth of HCT116 cells, and this process was suppressed by GEN-27 via inhibiting the secretion of proinflammatory cytokines IL-1*β* and IL-6 (Figure 3).

Inflammatory cytokines, growth factors, and chemokines, which are produced by inflammatory cells including macrophages, lymphocytes, or dendritic cells or, more often, by the tumor cells themselves, can regulate preneoplastic growth and the initiation of tumor, and they also play vital roles in two stages of tumor development: promotion and progression. For example, TNF plays a dual role in tumorigenesis. Low concentration of TNF can promote the development of inflammation-related cancers. On the other hand, TNF can disrupt tumor vasculature and induce cell apoptosis [3]. IL-6, as a multifunctional NF-*κ*B-regulated cytokine, is a critical tumor promoter during early CRC tumorigenesis via enhancing proliferation of tumor-initiating cells. IL-6 produced by lamina propria myeloid cells protects normal and premalignant intestinal epithelial cells (IECs) from apoptosis mediated by the transcription factor STAT3 [37]. Our previous study reported that oroxylin A, a natural flavonoid, inhibited colitis-associated carcinogenesis through modulating IL-6/STAT3 pathway in AOM/DSS mouse model and in HCT116 cells [38]. In AOM/DSS mice model, IL-1*β* levels in the colonic tissues are mainly produced by infiltrating neutrophils, prompt colon carcinogenesis by eliciting IL-17 response in intestinal myeloid cells [39]. These results indicated that inflammatory cytokines played an important role in inflammation-associated carcinogenesis. In this study, we found that GEN-27 treatment significantly decreased the excessive production of IL-6 and IL-1*β* in LPS-stimulated THP-1 cells in a dose-dependent manner without causing any cytotoxicity. The proliferation of HCT116 cells caused by conditional medium was significantly blocked by IL-1RA treatment, and the reduction caused by IL-1RA was further reduced by GEN-27, which suggested a vital role of IL-1*β* in GEN-27-mediated inhibitory action on cancer cell proliferation. These results also indicated that GEN-27 could potentially have preventive effect on colitis-associated CRC tumorigenesis.

Lipopolysaccharide (LPS), an endotoxin and the outer cell wall component of gram-negative bacteria, can trigger host inflammatory responses which are critical for host defense against bacterial infections [40]. LPS is specifically recognized by TLR4, a transmembrane receptor expressed in normal and malignant cells [41, 42]. The binding of LPS to TLR4 induces MyD88-dependent intracellular signaling. MyD88 recruits IL-1 receptor-associated kinases (IRAKs) and tumor-necrosis factor receptor-associated factor 6 (TRAF6) upon ligand stimulation, and then TRAF6 activates the transforming growth factor-*β*-activated kinase 1 (TAK1) complex. TAK1 then activates the IKK complex that mediates NF-*κ*B activation. Simultaneously, TAK1 activates the MAP kinase family, such as p38 MAPK, c-Jun NH_2_-termina kinase (JNK), and extracellular signal-regulated kinase (ERK) [43]. NF-*κ*B is an important transcription factor that controls cell survival by regulating programmed cell death, proliferation, and growth arrest, which is mediated by the downstream target genes. Activation of NF-*κ*B transcription factor is one of the main links between inflammation and tumorigenesis. Sustained activation of NF-*κ*B is found to be related to poor clinical prognosis of cancer. NF-*κ*B-driven cytokine production by myeloid cells is instrumental in CAC tumor growth, whereas NF-*κ*B activation in intestinal epithelial cells (IECs) promotes the survival of newly emerging premalignant cells [37]. Sustained activation of NF-*κ*B promotes growth of CRC by upregulating the antiapoptotic pathway and potentiating tumor cell survival [44]. It also enhances angiogenesis and invasiveness by mediating the production of cyclooxygenase 2 (COX-2), vascular cell adhesion molecule (VCAM), and matrix metalloproteinases (MMPs). Recent studies have shown that LPS-induced metastatic growth response depends on both TNF*α* production by host hematopoietic cells and NF-*κ*B activation in tumor cells. NF-*κ*B inhibition in colon cancer cells converts the LPS-induced growth response to tumor regression [45]. Genistein is reported to have significant inhibitory effect on NF-*κ*B pathway in many cancer cell lines [46–48]. Our data suggested that, similar with genistein, GEN-27 impaired the activation of NF-*κ*B pathway induced by LPS, which is demonstrated by the reduction in the translocation of NF-*κ*B p65 into nucleus and phosphorylation of I*κ*B*α* and IKK*α*/*β* both in THP-1 and in HCT116 cells (Figures 5 and 6).

IL-1*β* is a major proinflammatory cytokine with numerous roles in various physiological and pathological states. It also functions as a pleiotropic cytokine involved in tumor generation, growth, and metastasis in multiple types of cancers [49]. Recent studies have shown that IL-1*β* can promote sphere-forming capacity and EMT transformation concomitant with upregulated expression of stemness markers Bmi1 and nestin in colon cancer cells, suggesting that IL-1*β* may promote colon tumor growth and invasion through activation of CSC self-renewal and EMT [21, 50]. IL-1*β* was released from tumor-associated macrophages to activate WNT signaling and to promote the growth of tumor cells [20]. Our data showed that IL-1*β* significantly promoted cell growth in HCT116 cells, while this response could be inhibited by GEN-27 treatment, which is ascribed to the inactivation of NF-*κ*B pathway by GEN-27. It is suggested that GEN-27 could prevent IL-1*β*-induced cancer cell growth and could potentially be used as a chemopreventive agent against inflammation-related colon cancer. In fact, several anti-IL-1*β* agents have been tested in clinical trials in patients with diverse inflammatory diseases [51]. A better understanding of the intricate roles of IL-1*β* signaling in the malignant process will facilitate the application of novel IL-1*β* modulator in cancer patients.

In conclusion, we found that proinflammatory cytokines IL-6 and IL-1*β* were produced by LPS-stimulated THP-1 cells, which in turn promoted the proliferation of HCT116 cells. GEN-27 alone at low concentrations had no effect on the apoptosis or proliferation of HCT116 cells, but it significantly inhibited the growth of cancer cells in response to THP-1-conditioned medium through blocking NF-*κ*B signaling. In addition, GEN-27 remarkably suppressed IL-1*β*-mediated HCT116 cells proliferation, which confirmed the major role of IL-1*β* in promoting cancer cell growth. Our findings established that GEN-27 might serve as a potential chemopreventive agent against inflammation-associated colon cancer.
